# Disrupted functional connectivity of the emotion regulation network in major depressive disorder and its association with symptom improvement: A multisite resting-state functional MRI study

**DOI:** 10.1017/S0033291724003489

**Published:** 2025-02-05

**Authors:** Zhihui Lan, Lin-lin Zhu, You-ran Dai, Yan-kun Wu, Tian Shen, Jing-jing Yang, Ji-tao Li, Mingrui Xia, Xiaoqin Wang, Dongtao Wei, Bangshan Liu, Taolin Chen, Yanqing Tang, Qiyong Gong, Fei Wang, Jiang Qiu, Peng Xie, Lingjiang Li, Yong He, Yun-Ai Su, DIDA-MDD Working Group, Tianmei Si

**Affiliations:** 1 Peking University Sixth Hospital, Peking University Institute of Mental Health, NHC Key Laboratory of Mental Health (Peking University), National Clinical Research Center for Mental Disorders (Peking University Sixth Hospital), Beijing, China; 2 School of Mental Health, Wenzhou Medical University, Wenzhou, Zhejiang, China; 3 State Key Laboratory of Cognitive Neuroscience and Learning, Beijing Normal University, Beijing, China; 4 Beijing Key Laboratory of Brain Imaging and Connectomics, Beijing Normal University, Beijing, China; 5 IDG/McGovern Institute for Brain Research, Beijing Normal University, Beijing, China; 6 Department of Psychology, Southwest University, Chongqing, China; 7 Key Laboratory of Cognition and Personality (SWU), Ministry of Education, Chongqing, China; 8 Department of Psychiatry, The Second Xiangya Hospital, Central South University, Changsha, Hunan, China; 9 Mental Health Institute of Central South University, China National Clinical Research Center on Mental Disorders (Xiangya), China National Technology Institute on Mental Disorders, Hunan Technology Institute of Psychiatry, Hunan Key Laboratory of Psychiatry and Mental Health, Changsha, Hunan, China; 10 Huaxi MR Research Center (HMRRC), Department of Radiology, West China Hospital, Sichuan University, Chengdu, China; 11 Department of Psychiatry, The First Affiliated Hospital of China Medical University, Shenyang, China; 12 Institute of Neuroscience, Chongqing Medical University, Chongqing, China; 13 Chongqing Key Laboratory of Neurobiology, Chongqing, China; 14 Department of Neurology, The First Affiliated Hospital of Chongqing Medical University, Chongqing, China

**Keywords:** Antidepressant, Emotion regulation network, Functional connectivity, Major depressive disorder, Symptom improvement, RS-fMRI

## Abstract

**Background:**

The emotion regulation network (ERN) in the brain provides a framework for understanding the neuropathology of affective disorders. Although previous neuroimaging studies have investigated the neurobiological correlates of the ERN in major depressive disorder (MDD), whether patients with MDD exhibit abnormal functional connectivity (FC) patterns in the ERN and whether the abnormal FC in the ERN can serve as a therapeutic response signature remain unclear.

**Methods:**

A large functional magnetic resonance imaging dataset comprising 709 patients with MDD and 725 healthy controls (HCs) recruited across five sites was analyzed. Using a seed-based FC approach, we first investigated the group differences in whole-brain resting-state FC of the 14 ERN seeds between participants with and without MDD. Furthermore, an independent sample (45 MDD patients) was used to evaluate the relationship between the aforementioned abnormal FC in the ERN and symptom improvement after 8 weeks of antidepressant monotherapy.

**Results:**

Compared to the HCs, patients with MDD exhibited aberrant FC between 7 ERN seeds and several cortical and subcortical areas, including the bilateral middle temporal gyrus, bilateral occipital gyrus, right thalamus, calcarine cortex, middle frontal gyrus, and the bilateral superior temporal gyrus. In an independent sample, these aberrant FCs in the ERN were negatively correlated with the reduction rate of the HAMD_17_ score among MDD patients.

**Conclusions:**

These results might extend our understanding of the neurobiological underpinnings underlying unadaptable or inflexible emotional processing in MDD patients and help to elucidate the mechanisms of therapeutic response.

## Introduction

Major depressive disorder (MDD) is a highly prevalent psychiatric disorder characterized by symptoms of persistent low mood and decreased pleasure (Gnanavel & Robert, 2013). Impaired emotion regulation plays a putative role in the development and maintenance of depression (Donofry et al., 2016). Individuals with MDD often use inappropriate emotion regulation strategies, including more rumination and expressive suppression and less cognitive reappraisal (Aldao et al., 2010; Joormann & Vanderlind, 2014; Zhao et al., 2021b). Exploring the neuropathology underlying these depressed emotional states is expected to provide a theoretical basis and potential therapeutic targets for future precise treatment.

Although the neuropathological mechanisms of depression are not yet fully understood, many prior studies have suggested that emotion dysregulation may represent one of the core aspects of MDD (Park et al., 2019; Rive et al., 2013; Wu et a*l.*, 2022). Neuropsychological studies have identified several brain areas associated with emotion processing. Specifically, the ventrolateral prefrontal cortex (vlPFC) is involved in the inhibition of emotional appraisal (Aron et al., 2014); the amygdala is engaged in generating aversive emotions (e.g., fear and disgust) (Cisler et al., 2009; Kim et al., 2011); the anterior cingulate cortex (ACC) mediates sadness (Ramirez-Mahaluf et al., 2018); and the supplementary motor area (SMA) is implicated in both the cognitive and executive stages of emotion regulation (Kohn et al., 2014). Although the conclusions of these studies are not consistent, from the perspective of functional integration, emotional processing requires the collaborative activities of multiple brain regions (Morawetz et al., 2020).

Previous resting-state functional magnetic resonance imaging (RS-fMRI) studies identified 14 key brain regions constituting the emotion regulation network (ERN), including the amygdala, vlPFC, angular gyrus, SMA, precentral gyrus, and cingulate cortex, that were engaged in emotional processing (Kohn et al., 2014; Rey et al., 2016; Xu et al., 2020). The wide distribution of the ERN in the brain provides a framework for understanding the neuropathology of affective disorders. Numerous studies have reported functional aberrations in this network in patients with MDD (Kaiser et al., 2015; Li et al., 2022a; Ye et al., 2012), but the findings were inconsistent. For example, a previous RS-fMRI study demonstrated that patients with MDD exhibited reduced functional connectivity (FC) between the ACC and dorsolateral prefrontal cortex (dlPFC) compared to healthy controls (HCs) (Wang et al., 2016), while another study reported increased FC between the ACC and dlPFC in MDD patients (Ye et al., 2012). Furthermore, recent studies on antidepressant treatment have reported changes in emotion-related FC in patients with MDD after treatment. Xu et al. reported that MDD patients exhibited changed FC among the vlPFC, SMA, posterior cingulate cortex, and angular gyrus after electroconvulsive therapy, and these findings were correlated with clinical symptoms (Xu et al., 2020). Zhao et al. observed that after 12 weeks of antidepressant treatment, the MDD group exhibited altered dynamic FC among the amygdala, vlPFC, and cuneus compared to baseline (Zhao et al., 2021a). Despite these, due to the lack of large samples or cross-validated multicenter datasets, there is still no consensus on the whole-brain FC patterns of the ERN and their relationship to symptom improvement in patients with MDD. Delineating the functional connections of these brain regions associated with emotional processing may greatly advance our understanding of the neurobiological underpinnings underlying emotional dysregulation in MDD patients and help to elucidate the mechanisms of therapeutic response.

Here, we used a seed-based approach to examine whole-brain FC patterns in the ERN and their potential use as a neural biomarker for symptom improvement in patients with MDD. We first compared the FC in the ERN between the MDD and HC groups in a large cohort of the RS-fMRI dataset. Then, we explored the associations between the FC in the ERN and the efficacy of antidepressants in a separate dataset. Previous research has indicated that patients with MDD are commonly immersed in negative emotions and have a poor ability to modify emotions adaptively (Kassel et al., 2007). Thus, we first hypothesized that patients with MDD would show abnormal FC in the ERN. Secondly, we hypothesized that the FCs with between-group differences at baseline can predict the extent of symptom improvement.

## Materials and methods

### Participants

Two RS-fMRI datasets with strict quality control were included in this study. Dataset 1, composed of 1558 participants (782 MDD patients and 776 HCs), was obtained from five institutions in China (China Medical University, CMU; Central South University, CSU; Peking University Sixth Hospital, PKU; Sichuan University, SCU; Southwest University, SWU) through the Disease Imaging Data Archiving-Major Depressive Disorder Working Group (DIDA-MDD) (Xia et al., 2019). Dataset 2 included 45 first-episode drug-naïve patients (Supplementary Table 1) enrolled in the outpatient departments of Peking University Sixth Hospital. The patients in Dataset 2 received 8 weeks of antidepressant monotherapy with escitalopram, and the treatment outcomes were evaluated (see Supplemental Methods for details and additional assessments). All patients with MDD were diagnosed by experienced psychiatrists using the Mini-International Neuropsychiatric Interview (MINI) according to the Diagnostic and Statistical Manual of Mental Disorders, Fourth Edition (DSM-IV) criteria (Sheehan et al., 1998). On the day of the scan, the severity of the MDD symptoms was evaluated using the 17-item Hamilton Rating Scale for Depression (HAMD_17_) (Hamilton, 1967). The exclusion criteria included the following: (1) a history of brain injury; (2) any neurological disorders; (3) a concomitant major medical disorder; (4) pregnancy; (5) substance abuse or dependence; and (6) certain MRI-related contraindications. Phenotypical and imaging data underwent quality control checks for the following factors: the completeness of the clinical data and RS-fMRI scan, reading errors in the raw Digital Imaging and Communications in Medicine data, consistency of important scan parameters, incomplete anatomical brain images, excessive head movement, and coverage of the entire brain. The present study was approved by the ethics committees of each research center and was carried out in accordance with the Declaration of Helsinki. Prior to participating in the research, all participants provided written informed consent. The final sample in Dataset 1 consisted of 725 HCs and 709 patients with MDD (Table 1).

**Table 1.** Demographic and clinical characteristics of the participants

Center	Group	Age, mean (SD), years	Sex (M/F)	Education, mean (SD), years	Duration of illness,mean (SD), years	Medicated (Yes/No)	HAMD^a^, mean (SD)	Mean FD, mean (SD), mm
CMU, Shenyang	Healthy (n = 249)	27.24 (8.20)	103/146	14.85 (3.23)			1.10 (1.68)	.11 (.06)
	Patient (n = 125)	27.91 (9.70)	39/86	12.15 (3.07)	1.65 (3.17)	49/76	21.44 (8.67)	.11 (.07)
	Statistics T or χ^2^ /*p*	.70/.484	3.33/.068	7.72/<.001			33.71/<.001	1.07/.286
CSU, Changsha	Healthy (n = 108)	32.31 (7.96)	62/46	11.84 (3.40)			.62 (.88)	.13 (.06)
	Patient (n = 177)	36.28 (10.21)	77/100	10.16 (3.43)	2.52 (3.83)	N.A.	31.39 (7.82)	.14 (.07)
	Statistics T or χ^2^ /*p*	3.45/.001	5.19/.023	4.02/< .001			36.52/< .001	.88/.382
PKU, Beijing	Healthy (n = 73)	31.90 (9.01)	42/31	15.23 (2.28)				.18 (.07)
	Patient (n = 75)	31.51 (7.86)	44/31	13.76 (3.02)	.52 (.47)	0/75	25.35 (4.77)	.18 (.06)
	Statistics T or χ^2^ /*p*	.29/.775	.02/.889	3.39/.001				.91/.363
SCU, Chengdu	Healthy (n = 41)	34.83 (17.69)	17/24					.12 (.07)
	Patient (n = 50)	34.44 (12.90)	25/25	16.08 (4.22)	1.17 (1.60)	25/25	22.88 (4.25)	.11 (.07)
	Statistics T or χ^2^ /*p*	.12/.904	.66/.416					.71/.479
SWU, Chongqing	Healthy (n = 254)	39.65 (15.80)	88/166	12.80 (4.25)				.13 (.06)
	Patient (n = 282)	38.74 (13.65)	99/183	11.83 (3.72)	4.20 (5.52)	124/125	20.78 (5.88)	.13 (.05)
	Statistics T or χ^2^ /*p*	.72/.472	.01/.911	2.84/.005				1.68/.094

*Note*: SD, standard deviation; M, male; F, female; HAMD, Hamilton Rating Scale for Depression; FD, framewise displacement; CMU, China Medical University; CSU, Central South University; PKU, Peking University; SCU, Sichuan University; SWU, Southwest University.

a The CMU, PKU, SCU, and SWU research centers used the 17-item HAMD, while the CSU research center used the 24-item HAMD.

### Imaging acquisition and preprocessing

RS-fMRI data from all participants were gathered using gradient-echo planar imaging sequences on 3.0 T scanners. During the scan, the participants were instructed to close their eyes, unwind, avoid dozing off, stay still, and refrain from thinking about anything specific. The detailed acquisition parameters for each research center are listed in Supplementary Table 2. The RS-fMRI images were processed using the Data Processing Assistant for Resting-State fMRI (DPARSF, http://rfmri.org/DPARSF) software and custom code written in MATLAB. The detailed procedures included (1) removal of the first ten volumes (the first five volumes for the CSU research center due to the short scan time); (2) slice-timing correction for the remaining volumes; (3) realignment correction; 4) normalization into a stereotactic standard space (3 mm^3^ isotropic) using the EPI template; (5) spatial smoothing with a 6-mm full-width at half maximum Gaussian kernel; (6) detrending; (7) confounding covariate regression (including white matter, cerebrospinal fluid, and Friston-24 motion parameters); and 8) bandpass filtering (0.01–0.08 Hz). Finally, a “scrubbing” method was used to evaluate the impact of head motion on the RS-fMRI results (Power et al., 2012). The framewise displacement (FD) threshold was set at 0.5 mm, and linear interpolation was used to interpolate the signal at the fMRI volumes with excessive head motion. The FD values for each participant were calculated by applying the Jenkinson model (Jenkinson et al., 2002), which can characterize head motion within the scanner.

### Functional connectivity analyses of emotion regulation network

We performed FC analysis in the ERN according to the coordinated information from previous studies (Kohn et al., 2014; Morawetz et al., 2020). Because these four brain regions ((−42,22,-6) and (−34,27,-8), (−5,25,-10), and (0,50,1)) are anatomically close, we merged them and named them the left vlPFC and the left subgenual anterior cingulate cortex (sgACC), respectively. Finally, the remaining 14 regions (Supplementary Table 3) were used for the following analysis, including the bilateral angular gyrus (AG), bilateral amygdala (Amy), bilateral sgACC, bilateral vlPFC, bilateral precentral gyrus (preCG), left middle frontal cortex (MFC), posterior cingulate cortex (PCC), right inferior frontal gyrus (IFG), and SMA. FC was measured by computing the Pearson correlation coefficient between the mean blood oxygen level-dependent (BOLD) time series of all the voxels in each region of interest (ROIs) and the BOLD time series of other voxels in the rest of the brain. Using Fisher’s *r*-to-*z* transformation, the correlation coefficients were further *z*-transformed for standardization. Finally, center effects caused by scanners and sampling variations between centers were corrected using a ComBat model based on empirical Bayes (Johnson et al., 2007). Meanwhile, in order to preserve the effects of biological variables and avoid overcorrection, we included age, sex, and group as covariates (Xia et al., 2022).

### Statistical analyses

To test between-group differences, a two-sample *t-test* was used to compare the FC of ERN between the MDD patients and HCs with age, sex, and mean FD as covariates. The Gaussian random field (GRF) theory and Bonferroni correction were used to correct for multiple comparisons with the following threshold: voxel-wise *p* value <0.001 and cluster-wise *p* value <0.05/14.

To determine whether atypical FCs in the ERN can serve as measures of the prognostic effects of antidepressant therapy, we investigated potential associations between the FC in the ERN and the efficacy of antidepressants. In Dataset 1, for the FC maps of each seed, we defined clusters with between-group differences as masks. In Dataset 2, the mean FC values of the masks were extracted from the FC maps corresponding to the seeds for each participant. Partial correlation analyses were performed between the mean FC values and the reduction rate of the HAMD scores among the MDD patients, controlling for age, sex, and mean FD. The reduction rate of the HAMD score was used to evaluate the efficacy of the antidepressants and was calculated as follows: [(HAMD_baseline_- HAMD_8 weeks_) / HAMD_baseline_] × 100, where HAMD_baseline_ refers to the HAMD_17_ score before antidepressant treatment and HAMD_8 weeks_ refers to the HAMD_17_ score after antidepressant treatment for 8 weeks.

### Validation analyses

We repeated partial correlation analyses with the baseline HAMD as an additional covariate to further control for the effect of baseline depression severity on associations between the FC in the ERN and the efficacy of antidepressants (Supplementary Table 4). Moreover, we conducted a leave-one-site-out cross-validation methodology to ascertain that the results were not swayed by any particular site. Specifically, using a seed-based approach, we performed the inter-group comparisons five times, each time including four sites and leaving one site out (Supplementary Figure 1).

## Results

### Between-group differences in functional connectivity of ERN

**Figure 1.**
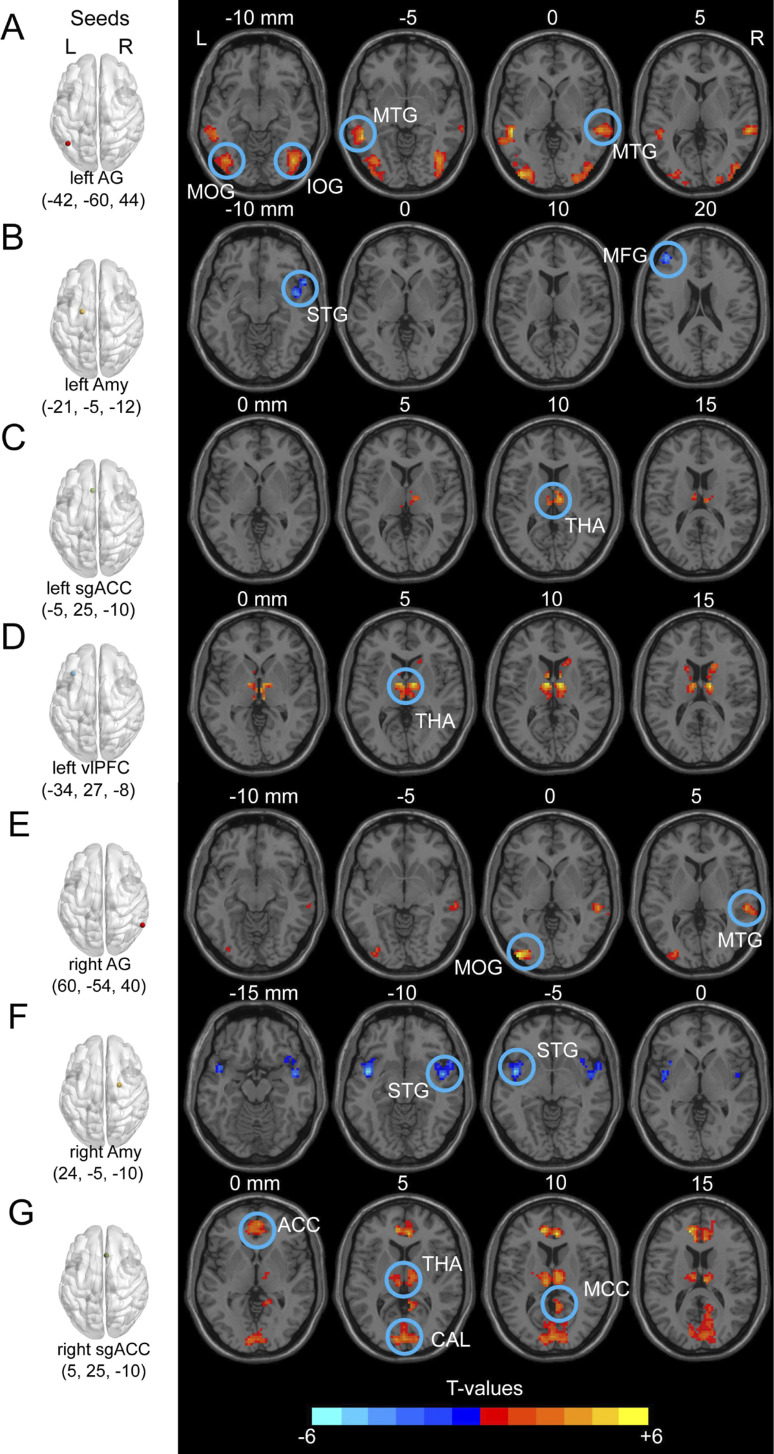
**Between-group comparisons of seed-based functional connectivity in the ERN between the MDD and HC groups (A-G).** The first column shows the seed regions. The brain regions (MOG, IOG, MTG, STG, MFG, THA, ACC, CAL, and MCC) that exhibited abnormal functional connectivity with the seed regions in patients with MDD compared with HCs are shown in the brain maps. L, left side; R, right side; AG, angular gyrus; MTG, middle temporal gyrus; MOG, middle occipital gyrus; IOG, inferior occipital gyrus; MFG, middle frontal gyrus; STG, superior temporal gyrus; Amy, amygdala; sgACC, subgenual anterior cingulate cortex; THA, thalamus; vlPFC, ventrolateral prefrontal cortex; ACC, anterior cingulate cortex; CAL, calcarine; MCC, middle cingulate cortex. All clusters were corrected for multiple comparisons with a voxel *p* < 0.001 and cluster *p* < 0.05/14 according to Gaussian random field theory and Bonferroni correction.

As shown in Figure 1 and Table 2, we observed significantly increased FC between the bilateral AG and bilateral middle temporal gyrus (MTG) as well as the bilateral occipital gyrus (Figure 1a and Figure 1e), the bilateral sgACC and right thalamus (THA) as well as the calcarine cortex (CAL, Figure 1c and Figure 1g), and the left vlPFC and right THA (Figure 1d) in MDD patients than in HCs. We also observed significantly decreased FC between the bilateral Amy and middle frontal gyrus (MFG) as well as the bilateral superior temporal gyrus (STG, Figure 1b and Figure 1F) in the MDD group compared to the HC group.

**Table 2.** Brain regions exhibiting group differences in emotion regulation network between the MDD and HC groups

Seed region	Brain region	Cluster size (voxels)	T value	Cluster-wise *p-value*	Peak MNI coordinates (mm)		
					X	Y	Z
Left AG	MDD > HC						
	Left MTG	173	3.086	0.002	−51	−36	0
	Right MTG	123	3.480	0.001	60	−36	3
	Left MOG	310	3.464	0.001	−39	−90	0
	Right IOG	455	3.578	<0.001	42	−72	−9
Left Amy	MDD < HC						
	Left MFG	83	−4.001	<0.001	−42	45	24
	Right STG	75	−2.958	0.003	48	0	−9
Left sgACC	MDD > HC						
	Right THA	99	5.058	<0.001	9	−15	12
Left vlPFC	MDD > HC						
	Right THA	345	3.329	0.001	9	−6	12
Right AG	MDD > HC						
	Left MOG	95	3.673	<0.001	−39	−90	0
	Right MTG	90	3.138	0.002	57	−33	6
Right Amy	MDD < HC						
	Left STG	150	−3.360	0.001	−45	3	−9
	Right STG	151	−3.225	0.001	48	0	−12
Right sgACC	MDD > HC						
	Left ACC	490	3.417	0.001	−12	42	12
	Right THA	198	5.021	<0.001	6	−18	12
	Left CAL	570	4.031	<0.001	0	−87	9
	Right MCC	134	3.758	<0.001	6	−36	36

*Note*: MNI, Montreal Neurological Institute; MDD, major depressive disorder; HC, healthy control; AG, angular gyrus; MTG, middle temporal gyrus; MOG, middle occipital gyrus; IOG, inferior occipital gyrus; MFG, middle frontal gyrus; STG, superior temporal gyrus; Amy, amygdala; sgACC, subgenual anterior cingulate cortex; THA, thalamus; vlPFC, ventrolateral prefrontal cortex; ACC, anterior cingulate cortex; CAL, calcarine; MCC, middle cingulate cortex.

### Relationships between functional connectivity in the ERN and antidepressant efficacy

**Figure 2.**
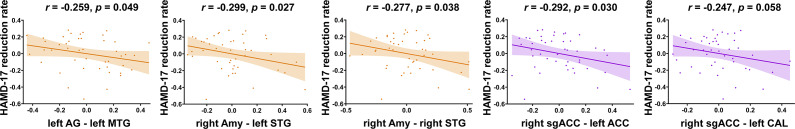
**Relationships between the functional connectivity aberrations in the ERN and the reduction rate of the HAMD_17_ score in patients with MDD.** The scatter maps show that the aberrant functional connectivity in the ERN was significantly correlated with the reduction rate of the HAMD_17_ score in the MDD group. HAMD_17_, 17-item Hamilton Rating Scale for Depression; AG, angular gyrus; MTG, middle temporal gyrus; STG, superior temporal gyrus; Amy, amygdala; sgACC, subgenual anterior cingulate cortex; ACC, anterior cingulate cortex; CAL, calcarine.

As shown in Figure 2, the FC between the left AG and left MTG (*r* = −0.258, *p*_uncorrected_ = 0.049), between the right Amy and left STG (*r* = −0.299, *p*_uncorrected_ = 0.027), between the right Amy and right STG (*r* = −0.277, *p*_uncorrected_ = 0.038), and between the right sgACC and left anterior cingulate cortex (ACC) (*r* = −0.292, *p*_uncorrected_ = 0.030) were negatively correlated with the reduction rate of the HAMD_17_ score in patients with MDD. There was a marginally significant negative correlation between the FC between the right sgACC and left CAL (*r* = −0.247, *p*_uncorrected_ = 0.058) and the reduction rate of the HAMD_17_ score in patients with MDD.

## Discussion

Using a seed-based FC approach, the present study investigated the whole-brain FC patterns of the ERN in the MDD and HC groups with a large-sample, multisite dataset. Compared to the HCs, patients with MDD exhibited aberrant FC between 7 ERN seeds and several cortical and subcortical areas, including the frontal gyrus, temporal gyrus, cingulate cortex, occipital gyrus, calcarine cortex, and THA. These aberrant FCs in the ERN were negatively correlated with the reduction rate of the HAMD_17_ score among MDD patients in an independent sample.

In this study, patients with MDD exhibited increased FC between the bilateral AG and bilateral MTG, and the FC between the left AG and left MTG was correlated with the extent of symptom improvement. The temporal cortex plays an important role in emotion, cognition, attention, and memory processing (Barbas, 2007; Haxby et al., 2001; Saxe, 2006; Saygin & Sereno, 2008). Numerous studies have demonstrated that many regions of the temporal cortex are altered in patients with MDD and are associated with antidepressant efficacy (Argyelan et al., 2016; Furtado et al., 2013; Hou et al., 2016; Qi et al., 2020; Wang et al., 2022). The MTG is located in the middle part of the temporal lobe and is a well-known structure that participates in the cognitive processing of emotions (Wang et al., 2021). There is a growing body of evidence that shows that MTG dysfunction is linked to depression. In addition, attention was given to the MTG not only in the cross-sectional study but also in the antidepressant intervention study of MDD. It has been found that antidepressant treatment changes FC between the left AG and left MTG in MDD patients (Mo et al., 2020), which was consistent with our results in Dataset 2. Therefore, the connection between the left AG and left MTG has the potential to serve as a biomarker for the antidepressant effects of drug treatment in patients with MDD. Moreover, we also found that MDD patients exhibited increased FC between the bilateral AG and the right IOG, and the left MOG. From a functional point of view, the IOG is implicated in the higher cognition of emotional processing, and the MOG is involved in the perception of emotional facial expression stimuli (Lan et al., 2021; Li et al., 2022b; Ma et al., 2023). Thus, dysfunction of the IOG and MOG may lead to clinical symptoms of emotional dysregulation in MDD patients to some extent. Together with our results, these findings indicate that FCs between the bilateral AG and the MTG, IOG, or MOG play a crucial role in the mechanism of development and treatment response in depression.

Moreover, we observed decreased FC between the Amy and MFG and increased FC between the left vlPFC and right THA in MDD patients, which provided evidence for the notion of a dysfunctional fronto-limbic neural loop in MDD (Drevets et al., 2008a; Johnstone et al., 2007; Klauser et al., 2015; Sindermann et al., 2021). The fronto-limbic neural loop includes frontal areas (cortical areas) and the limbic nucleus (Amy, THA, hippocampus, and striatum), which might be linked to top-down and bottom-up mechanisms in MDD, respectively. Consistent with our current findings, a previous review reported depression-specific structural and functional abnormalities in Amy and MFG during emotional tasks (Sindermann et al., 2021), and another RS-fMRI study revealed that MDD patients exhibited altered FC between the vlPFC and THA (Penner et al., 2018). The fronto-limbic neural loop contributes to depression-related disturbances in autonomic regulation and neuroendocrine responses via connections with visceral control structures (i.e., the hypothalamus and brainstem) (Drevets et al., 2008a). Therefore, the fronto-limbic neural loop might be a representative loop for elucidating the emotional dysregulation mechanisms underlying MDD.

Additionally, compared with HCs, increased FCs between the bilateral sgACC and the right thalamus (THA), left ACC, and calcarine cortex were found in the MDD group. More importantly, these FCs were correlated with the extent of symptom improvement. Increasing evidence indicates excessive glutamatergic afferents to sgACC lead to an increase in its activity, which is related to the pathophysiology of MDD (Drevets et al., 2008b; Liu et al., 2015; Mayberg et al., 2005; Morris et al., 2020). A reduction in sgACC activity has been consistently implicated in the neural mechanism of antidepressant treatment responses (Liu et al., 2015; Mayberg et al., 2005; Morris et al., 2020). Furthermore, the increased FC between the bilateral sgACC and right THA found in this study is also consistent with prior studies hypothesizing that increased FC between these two regions could exacerbate or sustain the negative emotional effects of chronic stress in in patients with MDD (Greicius et al., 2007; Hsu et al., 2014).

The results of dataset 2 revealed the negative correlation between abnormal FCs and the extent of symptom improvement, which indicated that the abnormality in the FCs at baseline can predict the efficacy of antidepressant treatment. Specifically, the patients with MDD exhibiting less extent of abnormal FCs may be more likely to benefit from the escitalopram treatment. These results demonstrate the potential of ERN’s abnormal FCs as biomarkers of antidepressant response.

There are several limitations to our present study. First, although we used a ComBat model to remove linear center effects brought on by scanners and sampling variations, potential nonlinear effects of these factors on brain imaging results might still exist. Thus, more advanced nonlinear models should be developed in future studies. Second, the sample size in Dataset 2 was relatively small, and further studies with larger sample sizes are needed to verify our findings. Third, due to the limited sample size of individuals undergoing antidepressant treatment, this is an exploratory and preliminary analysis. There may be potential relationships between the FC in the ERN and the efficacy of antidepressants in the MDD group, but the linear relationship is not strong. The results of the correlation analysis do not survive the correction for multiple comparisons, suggesting a need for future studies to verify our findings. Finally, the subtypes of MDD were not evaluated. Therefore, we were unable to compare the FC differences in the ERN across different subtypes.

## Conclusions

In conclusion, the current study illustrated widespread disrupted FC patterns in the ERN in patients with MDD using a large-sample, multisite dataset, and FCs with between-group differences were correlated with the extent of symptom improvement. Delineating these brain FC patterns might help to extend our understanding of the neurobiological underpinnings underlying unadaptable or inflexible emotional processing in MDD patients and to better identify the mechanism of therapeutic response.

## Supporting information

Lan et al. supplementary material Lan et al. supplementary material

## Data Availability

The analysis code and the data that support the findings of this study are available from the corresponding author upon reasonable request.

## Supplementary material

The supplementary material for this article can be found at http://doi.org/10.1017/S0033291724003489.
