# Supplementary material for: Disrupted functional connectivity of the emotion regulation network in major depressive disorder and its association with symptom improvement: A multisite resting-state functional MRI study
Source: Psychol Med. 2025 Feb 5;55:e21. doi: 10.1017/S0033291724003489 (PMC12017356; doi:10.1017/S0033291724003489)
Supplement: Lan et al. supplementary material [file S0033291724003489sup001.docx]

**Supplemental Material for:**

**Disrupted functional connectivity of the emotion regulation network in major depressive disorder and its association with symptom improvement: A multisite resting-state functional MRI study**

**Zhihui Lan^1#^, Lin-lin Zhu^1#^, You-ran Dai^1^, Yan-kun Wu^1^, Tian Shen^1^, Jing-jing Yang^2^, Ji-tao Li^1^, Mingrui Xia^3,4,5^, Xiaoqin Wang^6,7^, Dongtao Wei^6,7^, Bangshan Liu^8,9^, Taolin Chen^10^, Yanqing Tang^11^, Qiyong Gong^10^, Fei Wang^11^, Jiang Qiu^6,7^, Peng Xie^12,13,14^, Lingjiang Li^8,9^, Yong He^3,4,5^, Yun-Ai Su^1*^, DIDA-MDD Working Group, Tianmei Si^1*^**

**Supporting Document:**

**Supplemental Methods**

**Supplementary Table 1 Demographic and clinical characteristics of the MDD patients in Dataset 2 (n=45).**

**Supplementary Table 2 Scan parameters of the R-fMRI data from each center**

**Supplementary Table 3 The MNI central coordinates of the brain areas in the emotion regulation network.**

**Supplementary Table 4 Partial correlation analyses with the baseline HAMD as an additional covariate**

**Supplementary Figure 1 Leave-one-site-out cross-validation on seed-based functional connectivity in the ERN.**

**Materials and methods**

***Imaging dataset***

Dataset 2: Seventy-two first-episode drug-naïve patients with MDD were enrolled in the outpatient department of Peking University Sixth Hospital from October 2016 to December 2019. Experienced psychiatrists conducted psychiatric diagnoses of the MDD patients based on the Diagnostic and Statistical Manual of Mental Disorders, Fourth Edition (DSM-IV-TR) criteria. Depressive symptoms were evaluated using the 17-item Hamilton Rating Scale for Depression (HAMD_17_). The exclusion criteria were as follows: a concurrent comorbid Axis I disorder or Axis II personality disorder, pregnancy, substance dependence, acute suicide, intellectual disabilities, or MRI-related contraindications. This study was approved by the Ethics Committee of Peking University Sixth Hospital and was carried out in accordance with the Declaration of Helsinki. Before participating in this study, written informed consent was obtained from each participant.

Patients received 8 weeks of escitalopram treatment following baseline MRI scans. Among the initial 72 patients, 14 changed medications or discontinued treatment due to poor response or intolerable side effects, 11 patients refused to participate in further experiments because of unsuitable personal time, and 2 patients had excessive head movement. After these exclusions, 45 patients remained. The initial dose of escitalopram was 10 mg/day, increased to 20 mg/day within 1 week, and continued until the experiments were completed. During the study period, the dose of escitalopram for each patient was determined by the attending physician according to patient response. The final doses of escitalopram were 10 mg/day (n = 7), 15 mg/day (n = 14), or 20 mg/day (n = 24). At the end of the 8 weeks of treatment, the mean dose of escitalopram given to all patients was 16.9 ± 3.7 mg/day.

MRI data were gathered on a 3.0 T scanner (Siemens Magnetom Trio; Erlangen, Germany). Functional images were collected with an echo-planar imaging sequence (slice thickness = 4 mm, slice gap = 0.8 mm, 30 slices, repetition time (TR) = 2000 ms, echo time (TE) = 30 ms, flip angle (FA) = 90°, matrix = 64×64, and a total of 210 volumes). T1-weighted structural images were acquired with a magnetization-prepared rapidly acquired gradient-echo (MPRAGE) sequence (slice thickness = 1 mm, slice gap = 0 mm, TR = 2300 ms, TE = 3.01 ms, FA = 9°, matrix = 256 × 256). Before the scan, all participants were instructed to close their eyes, unwind, avoid dozing off, stay still, and refrain from thinking about anything specific. After the scan, no participant reported falling asleep.

Supplementary Table 1

Demographic and clinical characteristics of the MDD patients in Dataset 2 (n=45).

|  | Baseline | Week 8 |
| --- | --- | --- |
| Age (years) | 32.7±9.9 | 32.8±9.9 |
| Sex (male/female) | 17/28 |  |
| Education (years) | 15.5±2.1 |  |
| HAMD_17_ | 23.8±5.4 | 6.7±5.2 |
| Average dose of escitalopram (mg) |  | 16.9 ± 3.7 |

Note: Data are presented as mean ± standard deviation. HAMD_17_, 17-item Hamilton Rating Scale for Depression.

Supplementary Table 2

Scan parameters of RS-fMRI from each center

| Center | Scanner | TR  (ms) | TE  (ms) | FA  (°) | FOV  (mm^2^) | Matrix | Resolution  (mm^2^) | Slices | Thickness  (mm) | Gap  (mm) | VOL |
| --- | --- | --- | --- | --- | --- | --- | --- | --- | --- | --- | --- |
| CMU | GE HDxT 3T | 2000 | 40 | 90 | 240×240 | 64×64 | 3.75×3.75 | 35 | 3 | 0 | 200 |
| CSU | GE HDxT 3T | 2000 | 30 | 90 | 220×220 | 64×64 | 3.44×3.44 | 33 | 4 | 0.6 | 180 |
| PKU | Siemens Trio 3T | 2000 | 30 | 90 | 210×210 | 64×64 | 3.28×3.28 | 30 | 4 | 0.8 | 210 |
| SCU | GE EXCITE 3T | 2000 | 30 | 90 | 220×220 | 64×64 | 3.44×3.44 | 30 | 5 | 0 | 200 |
| SWU | Siemens Trio 3T | 2000 | 30 | 90 | 220×220 | 64×64 | 3.44×3.44 | 32 | 3 | 1 | 242 |

Abbreviations: TR, repetition time; TE, echo time; FA, flip angle; FOV, field of view; VOL, volume; CMU, China Medical University; CSU, Central South University; PKU, Peking University; SCU, Sichuan University; SWU, Southwest University; GE, General Electric.

Supplementary Table 3

MNI central coordinates of the brain areas in the emotion regulation network.

| **Seed regions** | **Abbreviation** | **MNI coordinates** | | |
| --- | --- | --- | --- | --- |
|  |  | **X Y Z** | | |
| Left angular gyrus | AG.L | -42 | -60 | 44 |
| Left amygdala | Amy.L | -21 | -5 | -12 |
| Left subgenual anterior cingulate cortex | sgACC.L | -5 | 25 | -10 |
| Left ventrolateral prefrontal cortex | vlPFC.L | -34 | 27 | -8 |
| Left precentral gyrus | preCG.L | -44 | 10 | 46 |
| Left middle frontal cortex | MFC.L | -38 | 22 | 44 |
| Posterior cingulate cortex | PCC | 0 | -56 | 20 |
| Right angular gyrus | AG.R | 60 | -54 | 40 |
| Right amygdala | Amy.R | 24 | -5 | -10 |
| Right inferior frontal gyrus | IFG.R | 50 | 30 | -8 |
| Right precentral gyrus | preCG.R | 48 | 8 | 48 |
| Right subgenual anterior cingulate cortex | sgACC.R | 5 | 25 | -10 |
| Right ventrolateral prefrontal cortex | vlPFC.R | 36 | 31 | -8 |
| Supplementary motor area | SMA | -2 | 14 | 58 |

Abbreviations: MNI: Montreal Neurological Institute; L, left side; R, right side.

Supplementary Table 4

Partial correlation analyses with the baseline HAMD as an additional covariate

| **Correlation** | ***r*** | ***p*-value** |
| --- | --- | --- |
| **The reduction rate of the HAMD_17_ score** |  |  |
| left AG - left MTG | -0.238 | 0.067 |
| right Amy - left STG | -0.282 | 0.037 |
| right Amy - right STG | -0.258 | 0.050 |
| right sgACC - left ACC | -0.299 | 0.029 |
| right sgACC - left CAL | -0.261 | 0.048 |


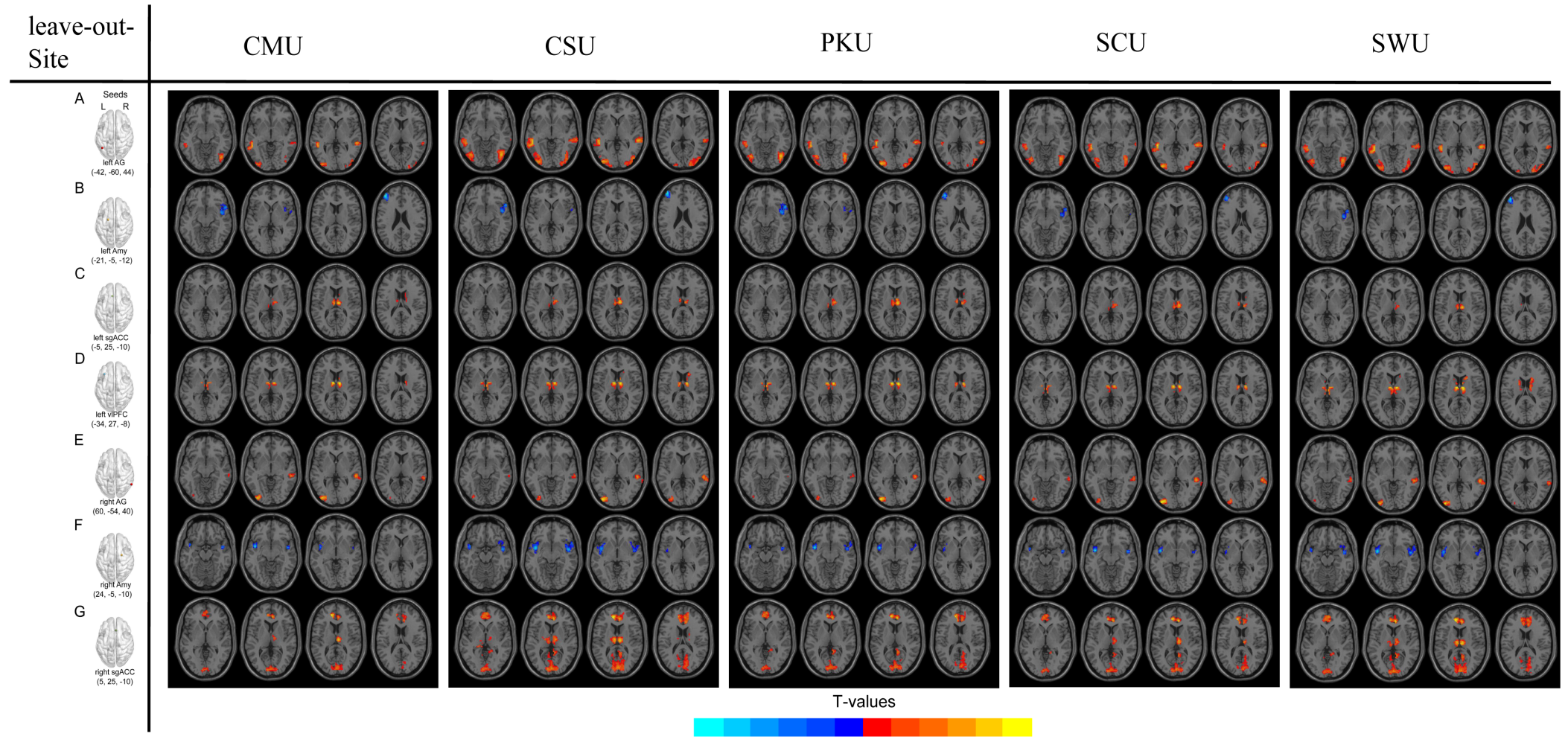


Supplementary Figure 1

Leave-one-site-out cross-validation on seed-based functional connectivity in the ERN. The inter-group difference connectivity in the Leave-one-site-out cross-validation methodology remained highly similar to our main findings (GRF: voxel-level *p* < 0.001, cluster-level *p* < 0.0035).
